# Coronary microvascular dysfunction as assessed by angiography-derived index of microvascular resistance co-localizes with and may explain the presence of ischemia in stress-cardiac magnetic resonance imaging in the absence of coronary artery disease

**DOI:** 10.3389/fcvm.2022.1060764

**Published:** 2022-11-24

**Authors:** Andrea Milzi, Rosalia Dettori, Richard Karl Lubberich, Kathrin Burgmaier, Nikolaus Marx, Sebastian Reith, Mathias Burgmaier

**Affiliations:** ^1^Department of Internal Medicine I, University Hospital RWTH Aachen, Aachen, Germany; ^2^Department of Pediatrics, University Hospital of Cologne, Cologne, Germany; ^3^Faculty of Applied Healthcare Science, Deggendorf Institute of Technology, Deggendorf, Germany; ^4^Department of Cardiology, Angiology and Electrophysiology, St. Franziskus-Hospital, Münster, Germany

**Keywords:** coronary artery disease, INOCA, myocardial ischemia, coronary physiology, index of microvascular resistance (IMR), coronary angiography, quantitative flow ratio (QFR)

## Abstract

**Introduction:**

Ischemia with no obstructive coronary disease (INOCA) is a frequent phenomenon in the cath lab. A possible cause is coronary microvascular dysfunction (CMD), which may be assessed by invasive testing with possible complications; therefore, less invasive approaches have emerged, such as the angiography-derived index of microvascular resistance (aIMR). The aim of our study was to investigate the association of single-vessel aIMR as a measure of CMD with areas of INOCA in stress testing.

**Methods:**

We measured aIMR in 286 vessels from 102 patients undergoing both stress cMRI and coronary angiography. Groups were (a) INOCA group (93 vessels, 32 patients); (b) coronary artery disease (CAD) control group (116 vessels, 42 patients) with ischemia due to relevant stenosis; and (c) control group (77 vessels, 28 patients) without ischemia or relevant stenosis.

**Results:**

INOCA patients presented higher mean aIMR (28.3 ± 5.7) compared to both CAD patients (17.4 ± 5.7, *p* < 0.001) and controls (22.1 ± 5.9, *p* < 0.001). Furthermore, in INOCA patients aIMR was significantly increased (33.0 ± 8.1 vs. 25.8 ± 6.3, *p* = 0.021) in vessels with vs. without ischemia. Single vessel aIMR presented a very good diagnostic efficiency in detecting INOCA [AUC 0.865 (0.804–0.925), optimal cut-off 27.1, *p* < 0.001].

**Conclusion:**

CMD, as assessed by 3-vessel aIMR, co-localizes with and may explain the presence of ischemia in stress-cMRI in INOCA.

## Introduction

An often found phenomenon in the cath lab is the presence of localized ischemia, e.g., in stress-cardiac magnetic resonance imaging (cMRI), in the absence of obstructive coronary artery disease (CAD) (ischemia with no obstructive CAD, INOCA). Coronary microvascular dysfunction (CMD) causes ischemia via a mismatch between demand and supply of blood flow due to structural and/or functional impairment of coronary microvessels (1). In order to cause ischemia, CMD does therefore not need obstruction of a major epicardial coronary vessel (1)—although CMD and obstructive CAD may coexist and contribute to patient symptoms. CMD may be assessed invasively by functional coronary angiography, which, however, bears patient risks due to pharmacological stimulation and wire advancement, as well as increased costs, radiation and procedural duration. Recently, approaches to assess CMD based only on coronary angiograms (angiography-derived index of microvascular resistance, aIMR) have emerged (2, 3).

Our hypothesis is that CMD as assessed by aIMR co-localizes with and explains ischemia in stress-cMRI in the absence of CAD; This was investigated in the present study.

## Materials and methods

This retrospective study included 286 vessels from 102 patients undergoing both coronary angiography and cMRI. Groups were:

(1) Patients with INOCA defined as localized ischemia in cMRI but no obstructive CAD (INOCA group, 93 vessels, 32 patients);
(2) Control patients with CAD, with ischemia in cMRI due to obstructive CAD (CAD group, 116 vessel, 42 patients);
(3) Control patients with neither ischemia in cMRI nor obstructive CAD (Control group, 77 vessel, 28 patients).

Clinical exclusion criteria were acute coronary syndromes, hemodynamic instability with use of vasopressors and/or assist devices, chronic total occlusions, arrhythmia, pregnancy, age < 18 years. Insufficient image quality (e.g., with excessive vessel overlap, insufficient vessel filling or lack of two suitable projections) was assessed case-by-case by researchers and led to study exclusion.

According to internal standard operating procedures, angiography was acquired at a minimum frame rate of 10 frames/second. Angiographies with lower frame rates were excluded from the analysis. Contrast dye injection was automatically performed by means of an ACIST injector. aIMR measurement was performed by an experienced, licensed cardiologist blinded to the clinical presentation of the patient and to the results of other tests (including cMRI). A commercially available software (QAngio XA 3D, Medis Medical Imaging System, Leiden, The Netherlands) was used to determine aIMR in all suitable vessels according to previously described protocols (4). As suggested (2), in a non-hyperemic state $a\,I\,M\,R=0.9^{*}\,P\,a^{*}\,Q\,F\,R^{*}\,\frac{V\,e\,s\,s\,e\,l\,L\,e\,n\,g\,t\,h}{V\,h\,y\,p\,e\,r\,e\,m\,i\,a}$, where *Vhyperemia* = 0.1 + 1.55*Vcontrast−0.93*Vcontrast^2^.

## Results

aIMR measurement was feasible in at least two coronary vessels in 102 patients of the initially 141 screened according to inclusion/exclusion criteria, with a success rate of 72.3%. Reasons for exclusion were poor image quality in 21 patients (53.8% of the exclusions), technical issues such as low frame rate or missing calibration in 11 (28.2%) and arrhythmia in 7 (17.9%).

**FIGURE 1:**
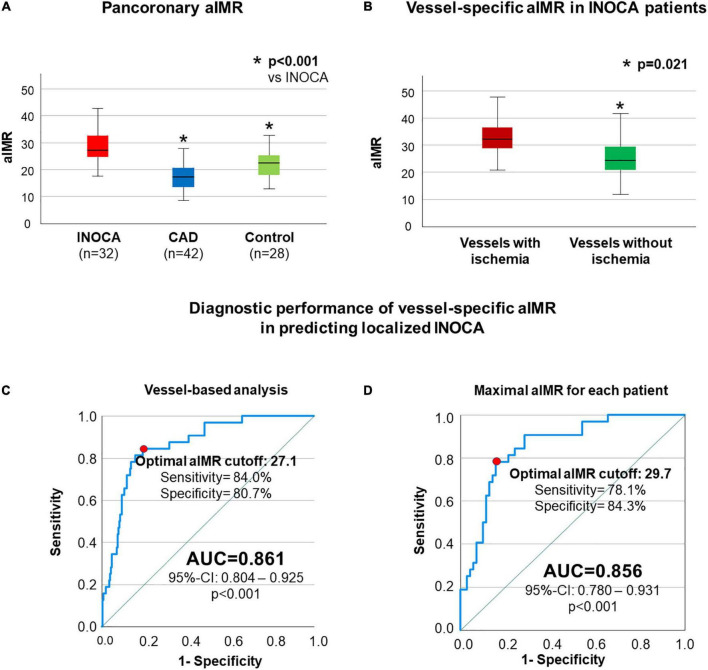
aIMR is higher in INOCA patients and vessels supplying ischemic myocardium. In **(A)** a significantly higher pancoronary aIMR in patients with INOCA compared to both CAD and control patients is shown. In **(B)** a comparison between single-vessel aIMR in INOCA patients is reported, showing significantly higher aIMR in vessels supplying ischemic myocardium compared to the rest of the coronary tree. ROC-analysis assessing vessel-specific aIMR in order to predict ischemia in cMRI in the supplied myocardium without obstructive CAD is shown in **(C**,**D)** including in the analysis all coronary vessels **(C)** or only the vessels with maximal aIMR for each patient **(D)**.

INOCA patients presented a highly significant increase in mean aIMR (28.3 ± 5.7) compared to both CAD patients (17.4 ± 5.7, *p* < 0.001) and controls (22.1 ± 5.9, *p* < 0.001, Figure 1A). In addition, to investigate if a localized increase in CMD as demonstrated by 3-vessel aIMR co-localizes with areas of ischemia in cMRI and might explain INOCA, we compared aIMR between vessels with and without ischemia in cMRI in INOCA patients. aIMR was significantly increased (33.0 ± 8.1 vs. 25.8 ± 6.3, *p* = 0.021, Figure 1B) in vessels with vs. without ischemia.

After we could demonstrate that elevated aIMR co-localizes with and therefore may explain ischemia as shown by cMRI, we next assessed the diagnostic efficiency of aIMR in predicting the presence of localized INOCA. Including all vessels in the analysis, single vessel aIMR presented a very good diagnostic efficiency in detecting this localized phenotype of INOCA [AUC 0.861 (0.804–0.925), optimal cut-off 27.1, *p* < 0.001, Figure 1C]. Similar results were obtained when only the maximal single vessel aIMR for each patient was used for the analysis [AUC 0.856 (0.780–0.931), optimal cut-off 29.7, *p* < 0.001, Figure 1D].

## Discussion

In this work we could demonstrate that CMD as determined by aIMR is significantly increased in INOCA patients compared to both, patients with obstructive CAD as well as control patients without ischemia. Furthermore, a localized increase in CMD as demonstrated by 3-vessel aIMR co-localizes with areas of ischemia in cMRI and thus may explain INOCA.

We are first to show that CMD as assessed with aIMR may explain ischemia as detected by stress cMRI in the absence of obstructive coronary disease. Our data is in line with previous studies which compared wire-based assessment of CMD with aIMR (2, 3). Our study extends the current knowledge by demonstrating that the often found phenomenon of the presence of ischemia in stress-cMRI in the absence of CAD co-localizes with and can be explained by CMD as assessed by aIMR. Therefore, in these INOCA patients the localized ischemia in cMRI derives from a relevant CMD rather than from an obstructive CAD; this is demonstrated by the elevated aIMR in comparison with the CAD group, where on the contrary the leading mechanism for ischemia is represented by a stenosis of epicardial vessels.

Furthermore, using aIMR we could detect the presence of INOCA and therefore validate aIMR as a wire- and medication-free method to determine CMD. Importantly, aIMR, compared to invasive functional coronary angiography, can measure CMD in all coronary arteries without repeated wire advancement or pharmacological stimulation and, therefore, without additional patient risk.

More interestingly, the co-localization of CMD as determined by an increased aIMR with areas of ischemia in cMRI generates hypotheses on the pathophysiology of such a localized INOCA phenotype. If CMD were solely the product of systemic risk factors, in fact, their effect would be diffuse and ubiquitous, similarly affecting all coronary vessels. Systemic risk factors, such as aging, hypertension, diabetes or nicotine use, definitely play a role in the pathogenesis of INOCA, as shown by their high prevalence in previous studies (5, 6). This is reflected by the overall elevated average aIMR in INOCA patients compared to both control groups in our study. However, this effect is not sufficient to explain our observations alone: the pronounced increase in aIMR in a single vessel territory and its association with localized ischemia may hint to local phenomena as possible causes. Previous studies, for instance, suggested that a low endothelial sheer-stress or paracrine effects may play a role in the genesis of INOCA (7), possibly by more heavily affecting a single vessel; this needs to be analyzed in further studies, but may shed further light on pathogenesis of CMD.

Despite being the first study to investigate the association of aIMR with localized ischemia in INOCA patients, the findings of our study need confirmation in larger, prospective studies. Although our data may pave the way to a more widespread use of non-invasive coronary physiology in the evaluation of CMD, further prospective studies are required to assess the clinical use of aIMR in guiding therapy of INOCA patients. Furthermore, due to study design and insufficient sample size, we are unable to draw any conclusion regarding prognosis and follow-up data is not available.

In conclusion, CMD as assessed by 3-vessel aIMR co-localizes with and may explain the presence of ischemia in stress-cMRI in the absence of CAD as an often found phenomenon in the cath lab.

## Data availability statement

The original contributions presented in this study are included in the article/supplementary material, further inquiries can be directed to the corresponding author/s.

## Ethics statement

The studies involving human participants were reviewed and approved by the EK 098/22 Ethikkommission RWTH Aachen. Written informed consent for participation was not required for this study in accordance with the national legislation and the institutional requirements.

## Author contributions

AM, RD, RL, SR, NM, and MB contributed to conception and design of the study. AM and RD performed the measurements. AM, RD, KB, and MB organized the database and performed the statistical analysis. AM wrote the first draft of the manuscript. All authors contributed to manuscript revision, read, and approved the submitted version.
